# Carotid-cavernous Fistula in a Patient with Minimal Head and Facial Trauma: A Case Report

**DOI:** 10.5811/cpcem.48556

**Published:** 2026-03-29

**Authors:** Yoshihiro Miyake, Tomohiro Abe, Keisuke Kubo, Hideki Nagoshi, Hidenobu Ochiai

**Affiliations:** * University of Miyazaki, Department of Emergency and Critical Care Medicine, Miyazaki, Japan; † Miyakonojo Medical Association Hospital, Department of Emergency and Critical Care Medicine, Miyazaki, Japan; ‡ Cardiovascular Biology Research Program, Oklahoma Medical Research Foundation, Oklahoma City, Oklahoma

**Keywords:** carotid-cavernous sinus fistula, delayed diagnosis, multiple trauma, case report

## Abstract

**Introduction:**

Intracranial arterial injury is typically associated with high-energy trauma. Early diagnosis and treatment are essential for improving the patient’s functional prognosis.

**Case Report:**

A 76-year-old woman complained of pulsatile tinnitus 15 days post motor vehicle collision. On presentation she was found to have sustained polytrauma, mostly to her abdominal and thoracic regions, as well as a facial contusion. She then developed ptosis, conjunctival congestion, and an ocular motility disorder in all directions in her right eye. Magnetic resonance angiography showed a direct high-flow shunt from the internal carotid artery to the cavernous sinus. Similar symptoms then developed on her left side. She underwent successful embolization to the left-sided fistula.

**Conclusion:**

Fistulization of the carotid with the cavernous sinus is a rare complication of head or facial trauma. Our case indicates that this can occur even in patients with seemingly minor head or facial trauma.

## INTRODUCTION

Carotid-cavernous fistula involves arteriovenous communication between the carotid artery and the cavernous sinus and is secondary to trauma in 70–90% of all cases.^1^ While it a rare complication of head and neck trauma, when present it is typically associated with high-energy trauma such as that involving a skull base fracture.^2^ The pathogenesis of carotid-cavernous fistula is markedly increased pressure in the cavernous sinus due to the influx of arterial blood into the cavernous sinus, followed by regurgitation and stasis of the ocular vein. The high pressure in the cavernous sinus damages the cranial nerves passing through the cavernous sinus (oculomotor, trochlear, ophthalmic, maxillary, and abducens nerves) causing various complex neuro-ophthalmic symptoms such as ptosis, conjunctival congestion, diplopia, and ocular motility disorders.^2^ In general, a direct shunt from the internal carotid artery into the cavernous sinus is characterized by higher shunt flow and more severe symptoms compared with indirect shunts.^3^

Early recognition and treatment are crucial for the preservation of cranial nerve function.^3^ Here we describe a case of direct carotid-cavernous fistula associated with minimal head and facial trauma. Despite its potentially treatable nature, this case illustrates the difficulty making the early diagnosis.

## CASE REPORT

**Image 1:**
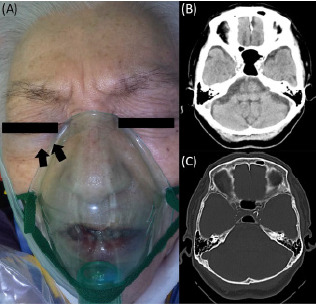
Facial appearance and cranial computed tomography (CT) performed on arrival. Mild periorbital ecchymoses was visible around the right eye (arrows) (A), and cranial CT shows no signs of cranial injury, skull fracture, or a carotid-cavernous fistula (B, C).

A 76-year-old woman with past medical history of hypertension and dyslipidemia experienced a motor vehicle collision. On arrival to the emergency department, her vital signs were as follows: Glasgow Coma Scale score, 14 (Eye 4, Verbal 4, Motor 6); blood pressure, 115/61 millimeters of mercury; heart rate, 58 beats per minute; respiratory rate, 24 per minute; and oxygen saturation, 100% (oxygen 6 liters per minute via mask). The patient complained of pain in the lumbar and pelvic regions. Mild periorbital ecchymoses were noted in her right eye (Image 1A). Pupils were equal and reactive to light, and no anisocoria was observed. No initial cranial nerve deficits were noted.

Computed tomography (CT) showed no intracranial hemorrhagic lesions and no fractures to the skull or facial bones (Images 1B, 1C). Other findings on CT included fractures of the left fourth and fifth ribs, a liver injury (the American Association for the Surgery of Trauma liver injury grade III), a right iliac fracture, a left lower abdominal subcutaneous hematoma, and a compression fracture of the first lumbar vertebra. Transcatheter arterial embolization was performed to treat the hemorrhage around the right iliac fracture. She was also transfused four units each of packed red blood cells and fresh frozen plasma.

**Image 2:**
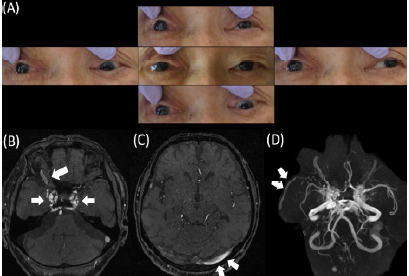
Ocular findings and magnetic resonance angiography (MRA) performed on Day 17: (A) Conjunctival congestion and an ocular motility disorder are evident in all directions of the right eye; (B) MRA showing bilateral cavernous sinuses and the right ophthalmic vein (arrows); and (C, D) The left transverse sinus and right middle cerebral vein (arrows) are also depicted.

**Image 3:**
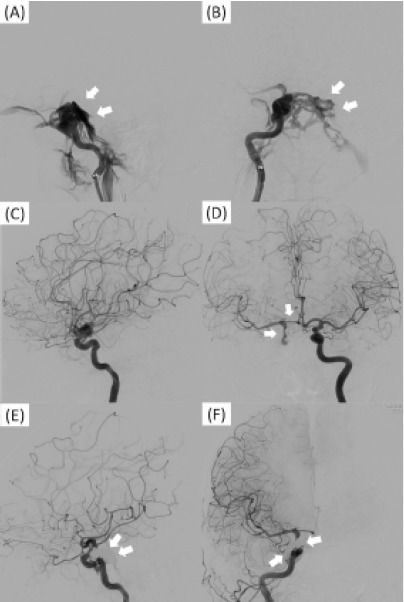
Angiography findings on Day 21 and after coil embolization on Day 23. (A)–(D) angiography on Day 21 and (E)–(F) after coil embolization on Day 23. (A), (C), and (E) show the lateral view. (B), (D), and (F) are the anteroposterior view.

Her general condition was stable, but on hospital day 15 swelling increased around her right eye and she complained of pulsatile tinnitus near her right eye. Two days later, she developed ptosis, conjunctival congestion, and an ocular motility disorder of the right eye (Image 2A). Magnetic resonance angiography (MRA) of the head showed shunting of the right internal carotid artery and cavernous sinus, and carotid-cavernous fistula was diagnosed (Images 2B-D). Three days later similar symptoms developed on the left. Contrast angiography of the right internal carotid artery showed contrast injection directly into the right cavernous sinus, confirming the exact location of the carotid-cavernous fistula, as well as regurgitation into the right superior ophthalmic vein and inferior ophthalmic vein. Regurgitation into the contralateral superior ophthalmic vein, inferior ophthalmic vein, and cortical vein via the intercavernous sinus was also evident (Images 3A, 3B). Contrast angiography of the left internal carotid artery showed no shunting into the cavernous sinus (Images 3C, 3D).

*CPC-EM Capsule*

What do we already know about this clinical entity?

*Carotid-cavernous fistula is a rare but serious complication, most often associated with severe face and head trauma*.

What makes this presentation of disease reportable?

*This case demonstrates a direct carotid-cavernous fistula developing in the subacute phase in a patient with minimal facial trauma*.

What is the major learning point?

*Even minor head or facial trauma may complicate a carotid-cavernous fistula in the subacute phase, hindering a timely diagnosis*.

How might this improve emergency medicine practice?

*Emergency physicians should consider carotid-cavernous fistula in patients presenting with neuro-ophthalmic symptoms, even after minor head or facial trauma*.

On Day 21, contrast angiography of the right internal carotid artery showed contrast influx from the internal carotid artery into the cavernous sinus (A) (white arrows) and reflux into the left cavernous sinus and cortical vein (B) (white arrows). Contrast angiography of the left internal carotid artery showed blood supply to both anterior arteries and the right middle cerebral artery with regurgitation into the right internal carotid artery (C, D) (white arrows), but no carotid-cavernous fistula directly into the cavernous sinus. After coil embolization on Day 23, contrast angiography of the right internal carotid artery showed disappearance of the shunt (E, F) (white arrows) and blood flow into the right middle cerebral artery.

The right carotid-cavernous fistula resolved post treatment noted on right internal carotid artery angiography (Images 3E, 3F). At the end of treatment, the neuro-ophthalmic symptoms in her left eye and the pulsatile tinnitus near the right eye resolved, but the symptoms in her right eye persisted. Although MRA on Day 27 showed resolution of the carotid-cavernous fistula on the right the ocular motility disorder, ptosis persisted.

## DISCUSSION

This case, in which the patient suffered a direct carotid-cavernous fistula during the subacute phase of trauma care, demonstrates the difficulty in making an early diagnosis of carotid-cavernous fistula because of the timing of its onset in a polytrauma patient. Treatment options include conservative management, surgery, and endovascular therapy. Spontaneous shunt closure is expected in only approximately 17% of direct carotid-cavernous fistula cases vs approximately 30% of indirect cases treated conservatively.^4^ Surgical interventions, such as suturing or clipping the fistula, packing the cavernous sinus, or ligating the internal carotid artery are invasive, although they can achieve shunt closure in 31–79% of carotid-cavernous fistula cases.^5^ Endovascular embolization of the shunt is the first choice of treatment, as it can successfully manage the fistula in more than 80% of cases with fewer adverse events compared with surgical intervention.^6^

Early diagnosis and treatment minimizes damage to the involved cranial nerves. The earlier the intervention the higher the chance of reversibility. Post shunt closure symptoms generally resolve within hours to days.^2^ Making the early diagnosis of carotid-cavernous fistula is challenging, particularly in the acute phase after trauma, when ocular findings may be subtle or overshadowed by other severe injuries. Moreover, patients with carotid-cavernous fistula are less likely to have typical symptoms: in fact, a systematic review reported that among patients with carotid-cavernous fistula, ptosis, conjunctival congestion, and diplopia occur in only 14%, 7%, and 2%, respectively.^7^ Furthermore, the onset of traumatic carotid-cavernous fistula varies widely, from immediately after injury to several weeks later, which adds complexity to the presentation.^8^ Our case adds to the literature that demonstrates carotid-cavernous fistula can develop in the subacute phase after injury and seemingly minor facial trauma.

Carotid-cavernous fistula is a rare complication. In this case the patient’s angiogram was delayed based on her evolving symptoms. However, the lesson from this case is not limited to carotid-cavernous fistula; in the management of severe trauma, delayed or missed diagnoses can lead to long-term disability. As seen in our case during the treatment of torso trauma, the diagnosis of non-fatal but complicated injuries can easily be delayed, particularly in patients with multiple trauma.^9^ Therefore, after addressing life-threatening conditions, careful and continued follow-up is essential to identify injuries that were not apparent during the initial assessment, and further diagnostic evaluation should be pursued as new symptoms emerge.

## CONCLUSION

The early diagnosis and treatment of carotid-cavernous fistula, a rare complication of head and facial trauma, can prevent neuro-ophthalmic symptoms. Carotid-cavernous fistula should be considered when neuro-ophthalmic symptoms are present after trauma, even in patients with minimal facial and/or head trauma, even in the subacute phase.
